# Impacts of metabolic disorders on short- and long-term mortality after coronary artery surgery in the elderly

**DOI:** 10.1186/s12872-022-02954-6

**Published:** 2022-11-24

**Authors:** Yuhong Fan, Jingjing Liu, Lei Jin, Zhonghe Liu, Lixiang Han, Yue Wang, Yangyang Zhang, Peiming Shen, Zhi Li

**Affiliations:** 1 Department of Cardiovascular Surgery, Shanghai Chest Hospital, Shanghai Jiao Tong University, 241 Huaihai Road, Shanghai, 200030 China; 2 Department of Cardiology, Wuxi People’s Hospital Affiliated to Nanjing Medical University, Wuxi, China; 3 Department of Critical Care Medicine, Shanghai Chest Hospital, Shanghai Jiao Tong University, 241 Huaihai Road, Shanghai, 200030 China; 4 Department of Cardiovascular Surgery, Jiangsu Province Hospital, The First Affiliated Hospital of Nanjing Medical University, 300 Guangzhou Road, Nanjing, 210029 China; 5 Outpatient Clinic, East Hospital, Tongji University School of Medicine, Shanghai, China

**Keywords:** Metabolic disorders, Coronary artery bypass grafting, Elderly, Mortality

## Abstract

**Background:**

Elderly patients undergoing cardiac operation often suffer various metabolic comorbidities, such as diabetes mellitus (DM) and obesity. The metabolic disorders in these individuals are widely considered to be possible predisposing factors for unfavourable prognosis. This retrospective study was aimed to determine the association of metabolic diseases with the mortality of elderly patients after coronary artery bypass grafting (CABG) and to identify the protective or risk factors related to their short- and long-term survival.

**Methods:**

Totally 684 patients aged 75 years or above undergoing isolated CABG were evaluated retrospectively. There were two groups depending on the body mass index (BMI): an overweight and obesity group (n = 354) and a normal weight and lean group (n = 330). Propensity score matching (PSM) was performed to adjust baseline clinical characteristics, which reduced confounding bias. The short-term postoperative mortality was tested via logistic regression. Kaplan–Meier and Cox regression analyses were done to compute the overall survival in each group and to identify relevant variables associated with all-cause mortality, respectively.

**Results:**

The prevalence rates of metabolic comorbidities in the total cohort were: diabetes mellitus (32.5%), overweight or obesity (51.8%) and hypertension (72.8%). The 30-day postoperative mortality was 5.1% and the long-term mortality was 15.25% at a median 46.2-month follow-up (1.0–178.6 months). The 30-day postoperative mortality was relevant to DM, diseased coronary arteries, New York Heart Association class, intra-aortic balloon pump and emergency surgery. The long-term mortality was negatively associated with overweight and obesity. Univariate and multivariate logistic regression recognized DM as an adverse factor related with 30-day postoperative mortality whether before or after PSM. The long-term mortality was not significantly relevant with DM (HR = 0.753, 95% CI 0.402–1.411). Overweight or obesity was not the risk factor of 30-day postoperative mortality (OR = 1.284, 95% CI 0.426–3.868), but was the protective factor of long-term survival (HR = 0.512, 95% CI 0.279–0.939).

**Conclusions:**

The “obesity paradox” exists regarding the prognosis of individuals aged ≥ 75, which was presented as lower long-term mortality no matter from all cause or cardio-cerebrovascular cause in patients with BMI ≥ 24.

*Trial registration* ChiCTR2200061869 (05/07/2022).

**Supplementary Information:**

The online version contains supplementary material available at 10.1186/s12872-022-02954-6.

## Background

The incidence of coronary atherosclerosis is climbing with age [1]. Coronary artery bypass grafting (CABG) is an important approach to treat complex coronary lesions and improve the quality of life. Elderly patients (75 years old and over) with coronary atherosclerosis who undergo CABG are often complicated with multiple age-related diseases. The higher risk of mortality after cardiac surgeries among elderly patients was reportedly associated with multiple primary metabolic diseases [2, 3].

For long time, obesity and diabetes mellitus (DM) are notorious for accelerating the progression and deteriorating the prognosis of various diseases. Losing weight and keeping normal glucose metabolism can significantly reduce the incidence of cardio-cerebrovascular events and improve the long-term prognosis after surgeries [4, 5]. However, researchers have noticed the “obesity paradox” in the prognosis after cardiac surgeries: neither short-term nor long-term mortality after CABG was adversely affected by obesity [6]. Moreover, patients with higher body mass index (BMI) have lower mortality after coronary artery surgeries [7].

However, most of these studies focused on the prognosis of white populations after cardiac surgeries at all ages. The elderly patients are quite different from the young patients in lifestyle, metabolism, and physiological functions. Furthermore, other coexisting metabolic diseases such as DM had been less studied in cooperation with overweight in the prognosis after cardiac surgeries. Therefore, this retrospective study aimed to observe and investigate the function of metabolic disorders and to identify the protective or risk factors for short- and long-term survival of elderly patients after isolated CABG in east China.

## Methods

### Patients

**Fig. 1:**
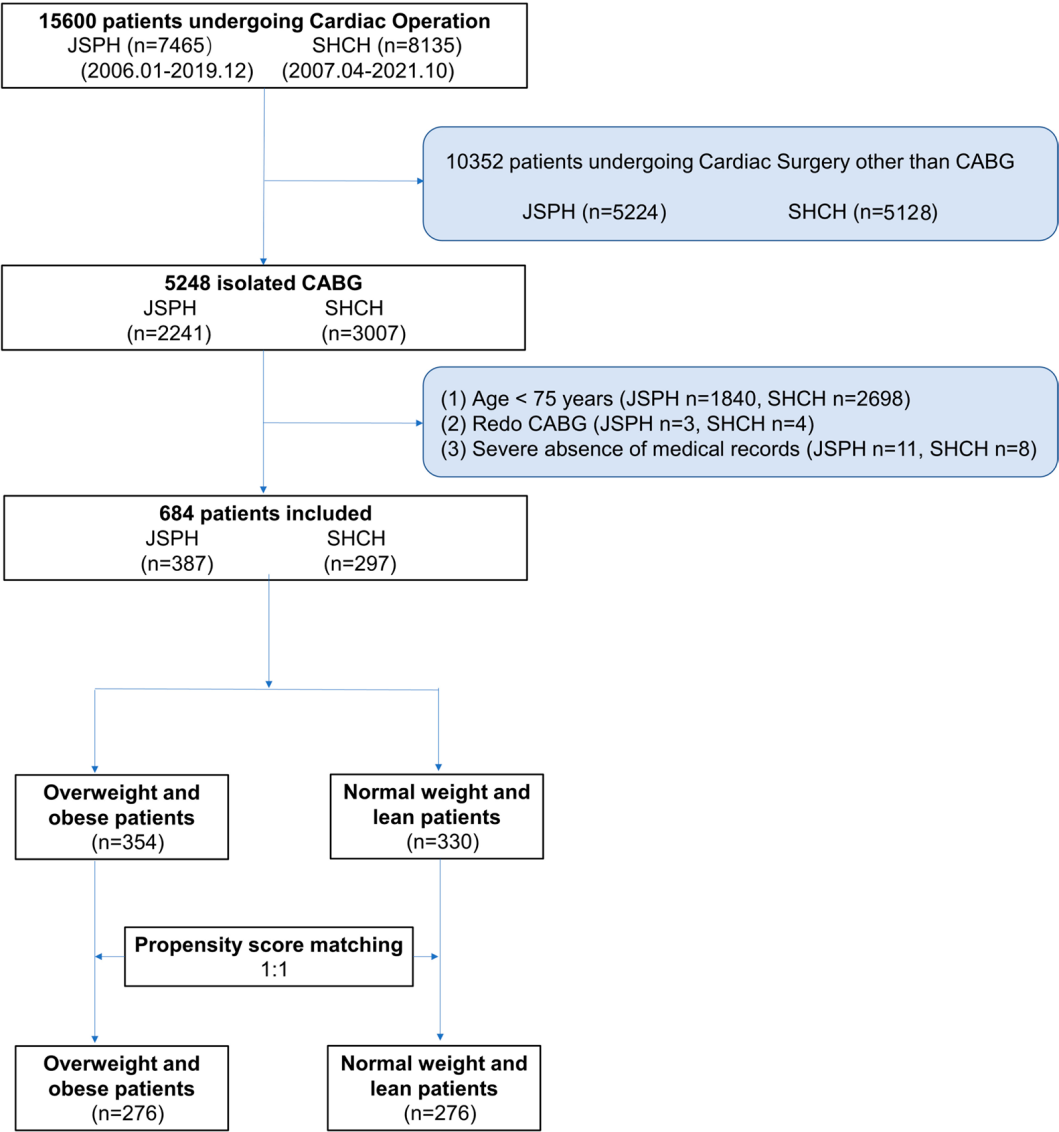
Patients’ enrollment flowchart. *CABG* coronary artery bypass grafting, *JSPH* Jiangsu Province Hospital, *SHCH* Shanghai Chest Hospital

**Fig. 2:**
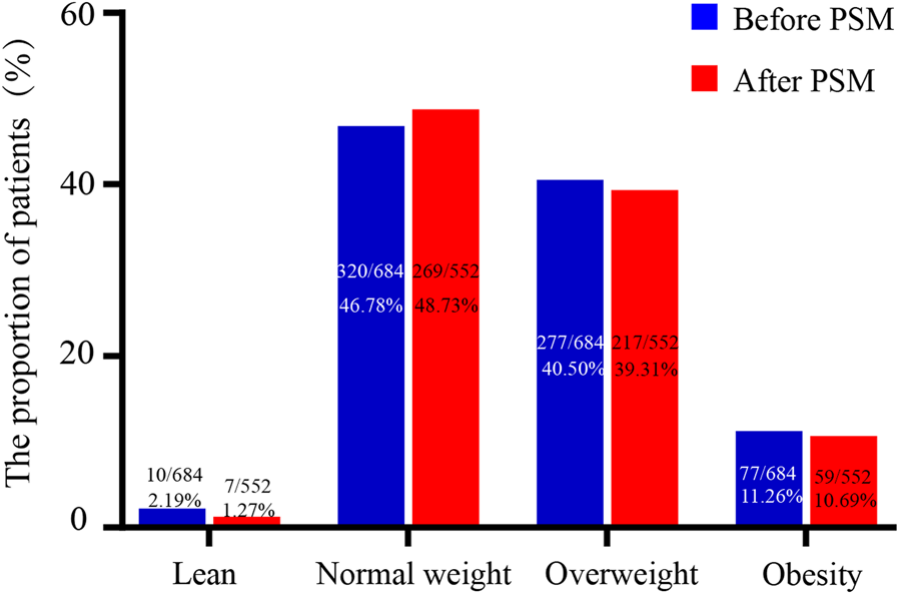
Different BMI distributions of the whole cohort before and after propensity score matching

This retrospective cohort study involved patients undergoing CABG from Jiangsu Province Hospital (JSPH) and Shanghai Chest Hospital (SHCH). From the initial 15,600 patients undergoing cardiac surgeries, 5248 patients receiving isolated CABG from the two hospitals were enrolled, including 2241 from JSPH and 3007 from SHCH. Among them, some patients were excluded for the following reasons: 1) age < 75 years old, 2) redo CABG, and 3) severe absence of medical records. Finally, 684 qualified patients were included. The detailed patients’ enrollment was presented in Fig. 1. According to the Chinese Society of Health Management and allied professional organizations recommended criteria with body mass index (BMI), BMI < 18, 18–23.9, 24–27.9 and ≥ 28 indicate lean, normal weight, overweight and obesity, respectively [8] (Fig. 2). Thus, 354 patients were classified into an overweight and obesity group, and the remaining 330 patients were involved into a normal weight and lean group.

This study did not involve patient treatment and ensured patients’ privacy, so individual consent was waived. The procedures conducted here were approved by the ethics committees of Jiangsu Province Hospital (2022SR209) and the ethics committees of the Shanghai Chest Hospital (IS22036). The clinical trial registration number was ChiCTR2200061869 (05/07/2022).

### Baseline characteristics

Demographic variables including sex, age, BMI, body surface area, types of coronary artery diseases (CADs), and comorbidities (DM, hypertension, cerebrovascular diseases (CVDs), peripheral vascular disease, preoperative atrial fibrillation, chronic pulmonary disease (COPD), pulmonary hypertension) were collected before operation. Types of CADs included stable angina pectoris, unstable angina pectoris and acute myocardial infarction. CVDs included stroke and lacunar infarction. Scores of the European System for Cardiac Operative Risk Evaluation II (EuroSCORE II) were calculated. Renal failure was defined as Ccr < 30 mL/min/1.73 m^2^. New York Heart Association (NYHA) grades were used to classify the cardiac function. The short-term postoperative mortality referred to the all-cause death within 30 days after surgery.

### Follow-up

Patients who survived after the operation were followed up according to the treatment routine. They were followed up at the first month, the third month, and every six months after discharge. If a patient had any change in his/her condition, he/she needed to seek nearby medical treatment in time. During the follow-up, if a patient died, the time and cause of death were recorded timely through family members. The last follow-up results were updated on March 25, 2022. The cardio-cerebrovascular (CCV) mortality included deaths from ischemic heart disease, chronic heart failure, hypertensive disease and cerebrovascular disease. Cerebrovascular disease included ischemic cerebrovascular disease, hemorrhagic cerebrovascular disease and certain complications following cerebrovascular disease. Ischemic heart disease included angina pectoris, myocardial infarction and subsequent complications following myocardial infarction. Hypertensive disease included hypertension and certain complications following hypertension.

### Statistical analysis

Kolmogorov–Smirnov (K–S) test was performed to assess the normal distribution of each variable. Continuous variables in normal distribution were expressed as mean ± standard deviation (SD), and categorical variables were presented as counts with percentages. Non-normally distributed variables were reported as medians and interquartile ranges. Wilcoxon rank-sum test was used for the comparison of continuous variables between 2 groups. Categorical variables were analyzed with Fisher’s exact or Chi-square test.

Clinical variables including age, gender, overweight and obesity, DM, hypertension, types of CADs, serum creatinine (Scr), renal failure, left ventricular ejection fraction (LVEF), COPD, and NYHA grade were estimated by univariate analysis to investigate the relationships with 30-day postoperative mortality. Then variables with *P* < 0.05 were analyzed by multivariate logistic regression to study the independent risk factors associated with 30-day postoperative mortality. Odds ratios (ORs) with 95% confidence intervals (CIs) were calculated to present binary outcomes. The Kaplan–Meier method was used to present patients’ survival, with the log-rank test to compare whether there was a significant difference in survival between the two groups. Variables like age, BMI, DM, hypertension, peripheral vascular disease, pulmonary hypertension, CVDs, previous PCI, CABG, male sex, types of CADs, preoperative CCR, COPD, preoperative atrial fibrillation and NYHA grade were taken into multivariate Cox regression to identify significantly different variables for long-term mortality. Hazard ratios (HR) were displayed with 95% CIs. The proportionality of hazards (PH) was assessed for each variable and Schoenfeld residuals were visually inspected for potential time-variant biases.

To minimize the bias of a retrospective observational study, we used 1:1 propensity score matching (PSM) to adjust preoperative variables between the two groups. Each patient with BMI ≥ 24 was matched to a patient with BMI < 24 by using variables: age, CADs classification, hypertension, LVEF, CVDs, pulmonary hypertension, intra-aortic balloon pump (IABP), and Euroscore II score. The optimal nearest neighbor matching algorithm without replacements was applied with the caliper width of 0.02. Cases lack of matched controls were excluded. The validity of PSM was evaluated by standard mean difference (SMD), and the covariate balance was presented with love plot (Additional file 2: Fig. S1). The absolute standardized difference < 0.2 was regarded as balanced matching. Totally 276 pairs of elderly patients were included in this propensity-matched cohort study. Univariate and multivariate logistic regression were applied to analyze the relationships of variates to 30-day postoperative mortality. Kaplan–Meier method and multivariate Cox regression were conducted to present patients’ survival and estimate significantly different variables for long-term mortality. Data processing for PSM and statistical analysis were performed with SPSS 22.0 (IBM, Chicago, IL, USA). *P* < 0.05 was considered to be significant.

Multi-collinearity diagnosis was performed with SPSS 22.0 (IBM, Chicago, IL, USA). The variance inflation factor (VIF) cutoff point < 10, tolerance > 0.1 and the Condition Index (CI) between 0 and 15 were considered as no collinearity. After collinearity analyzed of DM and overweight and obesity, we found no collinearity existed in these variables before and after PSM.

## Results

### Demographics and perioperative variables

The incidence rates of common chronic diseases attacking the elderly were high in both groups, such as hypertension, DM, COPD, pulmonary hypertension, peripheral vascular disease, and CVDs. The average age was 77.32 ± 2.71 in the normal weight and lean group, and was 76.59 ± 2.00 in the overweight and obesity group, with statistical difference (*P* = 0.014). The proportion of CVDs (24.58% vs. 15.76%, *P* = 0.009) and IABP implantation (5.37% vs. 3.03%, *P* = 0.028) were significantly higher in the overweight and obesity group. Moreover, the occurrence rates of hypertension (41.59% vs. 68.48%, *P* = 0.014), renal failure (1.69% vs. 5.76%, *P* = 0.005) and pulmonary hypertension (31.92% vs. 39.39%, *P* = 0.020) were significantly lower in the overweight and obesity group (Table 1).

**Table 1:** The baseline clinical characteristic of two groups before and after propensity score matching

	Before PSM				After PSM			
	Overweight and obese (n = 354)	Control group (n = 330)	*P* value	SMD (95% CI)	Overweight and obese (n = 276)	Control group (n = 276)	*P* value	SMD (95% CI)
Age(y)	76.59 ± 2.00	77.32 ± 2.71	0.014	− 0.308 (− 0.459; − 0.157)	76.76 ± 2.063	76.80 ± 2.276	0.764	− 0.020 (− 0.187; 0.147)
Male sex (n, %)	261 (73.73)	252 (76.36)	0.427	0.046 (− 0.104; 0.196)	210 (76.09)	216 (78.26)	0.544	0.052 (− 0.115; 0.219)
Weight (kg)	72.21 ± 8.38	59.67 ± 7.35	< 0.001	1.587 (1.415; 1.759)	72.42 ± 8.314	60.10 ± 7.221	< 0.001	1.583 (1.391; 1.774)
Height (cm)	164.51 ± 8.21	165.60 ± 7.47	0.150	− 0.139 (− 0.29; 0.01)	164.94 ± 7.928	166.01 ± 7.303	0.145	− 0.141 (− 0.308; 0.026)
BMI (kg/m^2^)	26.65 ± 2.14	21.70 ± 1.69	< 0.001	2.554 (2.352; 2.756)	26.58 ± 2.06	21.75 ± 1.65	< 0.001	2.592 (2.366; 2.819)
BSA (m^2^)	1.89 ± 0.15	1.74 ± 0.13	< 0.001	1.079 (0.919; 1.240)	1.89 ± 0.14	1.75 ± 0.13	< 0.001	1.083 (0.904; 1.261)
Types of CAD			0.030	− 0.166 (− 0.317; − 0.016)			0.618	− 0.042 (− 0.209; 0.124)
Stable angina pectoris (n, %)	106 (29.94)	83 (25.15)			78 (28.26)	76 (27.54)		
Unstable angina pectoris (n, %)	217 (61.30)	201 (60.91)			171 (61.96)	168 (60.87)		
Acute myocardial infarction (n, %)	31 (8.76)	46 (13.94)			27 (9.78)	32 (11.59)		
Diabetes mellitus (n, %)	122 (34.46)	100 (30.30)	0.246	0.085 (− 0.065; 0.235)	93 (33.70)	78 (28.26)	0.168	0.118 (− 0.049; 0.285)
Hypertension (n, %)	272 (41.59)	226 (68.48)	0.014	0.203 (0.053; 0.353)	202 (73.19)	195 (70.65)	0.508	0.056 (− 0.111; 0.223)
NYHA classification			0.566	− 0.047 (− 0.197; 0.103)			0.224	0.104 (− 0.063; 0.271)
I (n, %)	12 (3.39)	12 (3.64)			8 (2.90)	12 (4.35)		
II (n, %)	193 (54.52)	172 (52.12)			148 (53.62)	156 (56.52)		
III (n, %)	136 (38.42)	131 (39.70)			108 (39.13)	98 (35.51)		
IV (n,%)	13 (3.67)	15 (4.55)			12 (4.35)	10 (3.62)		
LVEF (%)	60.09 ± 7.70	58.69 ± 8.93	0.137	0.167 (0.017; 0.318)	59.25 ± 7.73	59.80 ± 8.30	0.096	− 0.068 (− 0.235; 0.099)
Cerebrovascular disease			0.009	0.200 (0.049; 0.350)			0.401	− 0.072 (− 0.238; 0.095)
Stroke (n, %)	53 (14.97)	31 (9.39)			14 (5.07)	21 (7.61)		
Lacunar infarction (n, %)	34 (9.60)	21 (6.36)			33 (11.96)	30 (10.87)		
Preoperative Scr (mmd/L)	87.28 ± 25.30	87.51 ± 37.13	0.306	− 0.007 (− 0.157; 0.143)	87.58 ± 24.58	87.02 ± 36.50	0.102	0.018 (− 0.149; 0.185)
Peripheral vascular disease (n, %)	35 (9.89)	35 (10.61)	0.757	− 0.033 (− 0.183; 0.117)	29 (10.51)	26 (9.42)	0.671	0.036 (− 0.131; 0.203)
Pulmonary hypertension (n, %)	241 (31.92)	200 (39.39)	0.020	− 0.179 (− 0.329; − 0.029)	94 (34.06)	91 (32.98)	0.899	0.011 (− 0.156; 0.178)
Renal failure (n, %)	6 (1.69)	19 (5.76)	0.005	− 0.214 (− 0.365; − 0.064)	4 (1.45)	9 (3.26)	0.161	− 0.119 (− 0.286; 0.048)
Preoperative atrial fibrillation (n, %)	13 (3.67)	17 (5.15)	0.346	− 0.049 (− 0.199; 0.101)	12 (4.35)	12 (4.35)	1.000	0.000 (− 0.167; 0.167)
COPD (n, %)	26 (7.34)	30 (9.09)	0.406	− 0.073 (− 0.223; 0.077)	24 (8.70)	21 (7.61)	0.641	0.040 (− 0.127; 0.207)
Valvular disease (n, %)	45 (12.71)	39 (11.82)	0.722	0.030 (− 0.120; 0.180)	38 (13.77)	35 (12.68)	0.707	0.032 (− 0.135; 0.199)
Previous PCI (n, %)	39 (11.02)	40 (12.12)	0.652	− 0.031 (− 0.181; 0.119)	35 (12.68)	27 (9.78)	0.282	0.092 (− 0.075; 0.259)
Diseased Coronary arteries	2.79 ± 0.49	2.81 ± 0.49	0.422	− 0.041 (− 0.191; 0.109)	2.76 ± 0.49	2.83 ± 0.46	0.024	− 0.145 (− 0.312; 0.022)
IABP implantation (n, %)	19 (5.37)	10 (3.03)	0.028	0.174 (0.024; 0.324)	7 (2.54)	6 (2.17)	0.666	0.037 (− 0.130; 0.204)
Cardiopulmonary bypass (n, %)	30 (8.47)	25 (7.58)	0.666	0.000 (− 0.150; 0.150)	22 (7.97)	19 (6.88)	0.627	0.041 (− 0.125; 0.208)
Coronary artery bypass grafts	3.30 ± 0.90	3.26 ± 0.93	0.659	0.044 (− 0.106; 0.194)	3.26 ± 0.90	3.29 ± 0.96	0.806	0.020 (− 0.147; 0.186)
Emergency operation (n, %)	15 (4.24)	7 (2.12)	0.116	0.091 (− 0.059; 0.241)	13 (4.71)	5 (1.81)	0.038	0.177 (0.010; 0.344)
EuroSCORE II	3.08 ± 2.64	3.56 ± 3.61	0.102	− 0.152 (− 0.302; − 0.002)	3.16 ± 2.69	3.08 ± 2.52	0.676	0.030 (− 0.137; 0.197)
30-day postoperative mortality (n, %)	17 (4.80)	18 (5.45)	0.699	0.000 (− 0.150; 0.150)	12 (4.35)	8 (2.90)	0.363	0.077 (− 0.090; 0.244)

*PSM* propensity score matching, *SMD* standard mean difference, *BMI* body mass index, *BSA* body surface area, *CAD* coronary artery disease, *NYHA* New York heart association, *LVEF* left ventricular ejection fraction, *Scr* serum creatinine, *COPD* chronic obstructive pulmonary disease, *PCI* percutaneous coronary intervention, *IABP* intra-aortic balloon pump, *EuroSCORE II* European system for cardiac operative risk evaluation II

After PSM, the 276 matched pairs showed no significant difference in age, CADs types, LVEF, EuroSCORE II, or the occurrence of pulmonary hypertension, renal failure, hypertension and CVDs. The numbers of coronary lesion vessels and bypass grafts were generally similar between groups. Besides, the accompanied cardiac diseases such as valvular disease or preoperative atrial fibrillation were not significantly different between groups. Both groups involved a higher proportion of men. Excepted for weight, BSA and BMI, the remaining covariates were balanced matched between the groups with the absolute SMD < 0.2. No significant difference was found in 30-day postoperative mortality between two groups whether before or after PSM (Table 1).

### 30-day postoperative mortality

Among the 684 patients, 5.1% died within 30 days postoperatively. The 30-day mortality after CABG in the overweight and obesity group versus the normal weight and lean group was 4.80% vs. 5.45%. DM (OR = 2.502, 95% CI 1.116–5.613, *P* = 0.026), preoperative Scr (OR = 1.011, 95% CI 1.003–1.019, *P* = 0.008), NYHA classification (OR = 2.701, 95% CI 1.395–5.231, *P* = 0.003), IABP (OR = 3.373, 95% CI 1.979–5.749, *P* < 0.001) and emergency operation (OR = 6.179, 95% CI 2.340–16.316, *P* < 0.001) were significantly related to the increased risk of 30-day postoperative mortality (Table 2).

**Table 2:** Risk factors of 30-day postoperative mortality by univariate and multivariate logistic regression analysis before and after propensity score matching

	Before PSM				After PSM			
	Univariate logistic regression		Multivariate logistic regression		Univariate logistic regression		Multivariate logistic regression	
	OR	P value	OR	*P* value	OR	P value	OR	*P* value
Age	1.116 (0.990–1.258)	0.074	1.091 (0.941–1.265)	0.249	1.005 (0.819–1.232)	0.965	0.920 (0.680–1.245)	0.590
Male sex	1.041 (0.478–2.267)	0.920	1.016 (0.394–2.642)	0.974	0.840 (0.276–2.560)	0.759	0.640 (0.150–2.723)	0.546
Overweight and Obese	0.874 (0.443–1.727)	0.699	0.677 (0.289–1.588)	0.370	1.523 (0.613–3.785)	0.365	1.284 (0.426–3.868)	0.657
Diabetes mellitus	2.046 (1.033–4.051)	0.040	2.502 (1.116–5.613)	0.026	2.842 (1.155–6.991)	0.023	3.218 (1.062–9.752)	0.039
Hypertension	1.083 (0.498–2.357)	0.840			1.585 (0.522–4.819)	0.417		
Cerebrovascular disease	0.839 (0.451–1.563)	0.581			1.360 (0.688–2.687)	0.376		
LVEF	0.945 (0.915–0.976)	0.001	0.967 (0.926–1.009)	0.120	0.941 (0.902–0.981)	0.005	0.959 (0.901–1.021)	0.188
NYHA classification	2.690 (1.587–4.558)	0.000	2.701 (1.395–5.231)	0.003	4.184 (2.079–8.422)	< 0.001	5.391 (2.072–14.026)	0.001
Types of CAD	1.154 (0.657–2.029)	0.618			1.425 (0.676–3.005)	0.352		
Scr	1.013 (1.006–1.020)	< 0.001	1.011 (1.003–1.019)	0.008	1.014 (1.005–1.022)	0.002	1.015 (1.003–1.027)	0.013
Peripheral vascular disease	1.140 (0.390–3.329)	0.811			1.629 (0.462–5.744)	0.448		
Pulmonary hypertension	1.587 (1.024–2.459)	0.039	0.835 (0.468–1.490)	0.542	1.044 (0.541–2.016)	0.898	0.398 (0.154–1.029)	0.057
Preoperative atrial fibrillation	2.160 (0.622–7.497)	0.225			2.576 (0.562–11.800)	0.223		
COPD	1.054 (0.312–3.558)	0.932			1.264 (0.284–5.628)	0.759		
Previous PCI	2.412 (1.055–5.573)	0.037	1.055 (0.361–3.082)	0.922	1.415 (0.403–4.972)	0.588	0.570 (0.099–3.296)	0.530
Valvular disease	1.203 (0.453–3.190)	0.711			1.165 (0.333–4.077)	0.811		
IABP	3.255 (2.129–4.976)	< 0.001	3.373 (1.979–5.749)	< 0.001	3.492 (1.837–6.638)	< 0.001	3.749 (1.546–9.089)	0.003
Diseased Coronary arteries	0.620 (0.355–1.081)	0.092			0.687 (0.314–1.504)	0.348		
Emergency operation	5.591 (2.456–12.726)	< 0.001	6.179 (2.340–16.316)	< 0.001	4.944 (1.719–14.222)	0.003	7.413 (1.975–27.822)	0.003
Coronary artery bypass grafts	0.816 (0.566–1.178)	0.279			0.806 (0.501–1.297)	0.374		

*PSM* propensity score matching, *OR* odd ratio, *LVEF* left ventricular ejection fraction, *NYHA* New York heart association, *CAD* coronary artery disease, *Scr* serum creatinine, *COPD* chronic obstructive pulmonary disease, *PCI* percutaneous coronary intervention, *IABP* intra-aortic balloon pump

After PSM, DM (OR = 3.218, 95% CI 1.062–9.752, *P* = 0.039), NYHA classification (OR = 5.391, 95% CI 2.072–14.026, *P* = 0.001), Scr (OR = 1.015, 95% CI 1.003–1.027, *P* = 0.013) and emergency operation (OR = 7.413, 95% CI 1.975–27.822, *P* = 0.003) were independent risk factors for 30-day postoperative mortality in the elderly. IABP, an effective circulation assistance device to treat the low cardiac output syndrome, is usually applied in high-risk patients. IABP implantation was positively correlated with the 30-day postoperative mortality in the elderly (OR = 3.749, 95% CI 1.546–9.089, *P* = 0.003). The univariate analysis and multivariable logistic regression showed no correlation between BMI and surgical mortality in the elderly (Table 2).

### Long-term mortality

**Fig. 3:**
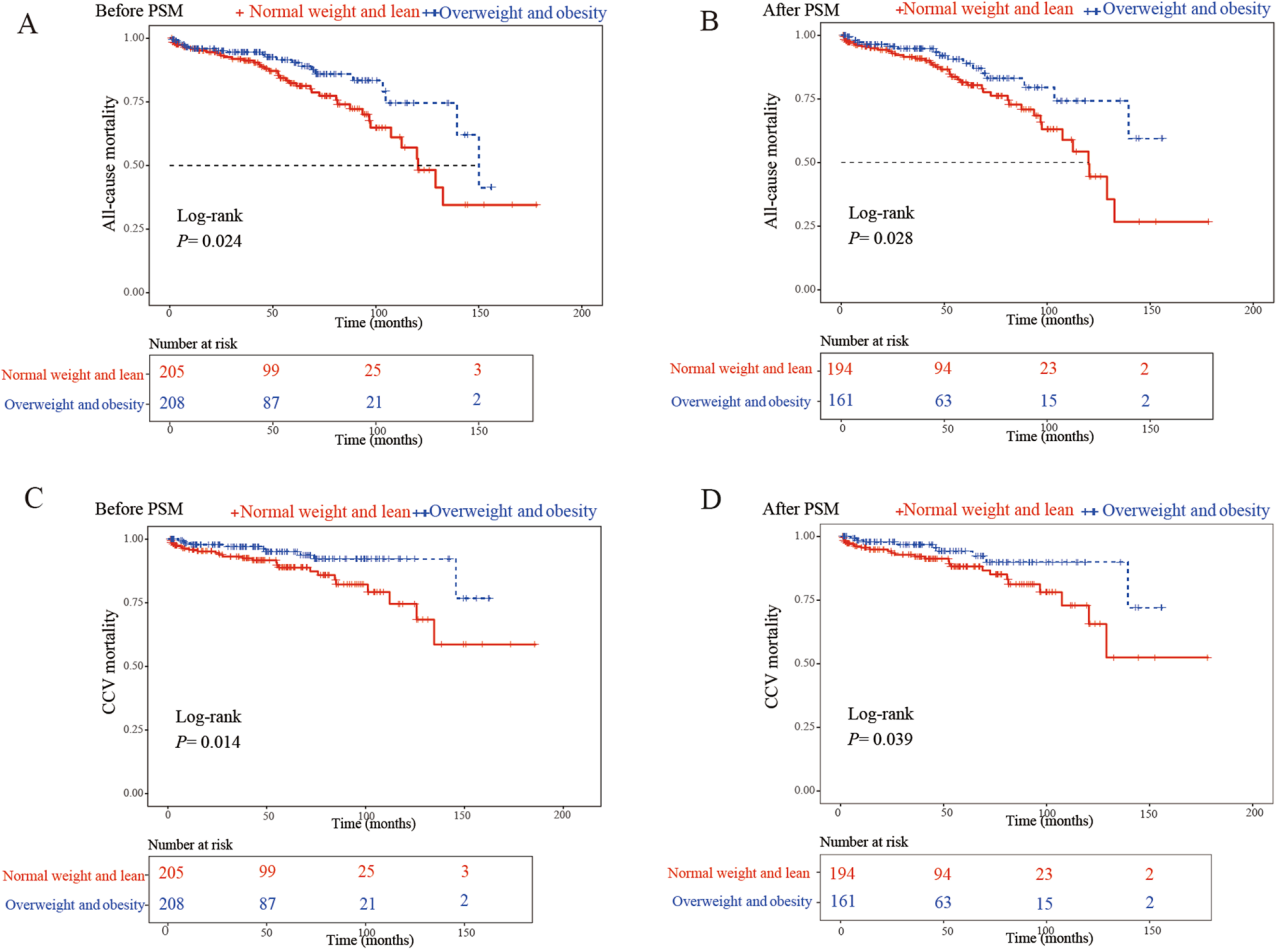
Kaplan–Meier curves for mortality up to 178.6 months in two groups divided by BMI categories. Kaplan–Meier curves of all-cause mortality before (**A**) and after (**B**), and of cardio-cerebrovascular mortality before (**C**) and after (**D**) propensity score matching

**Fig. 4:**
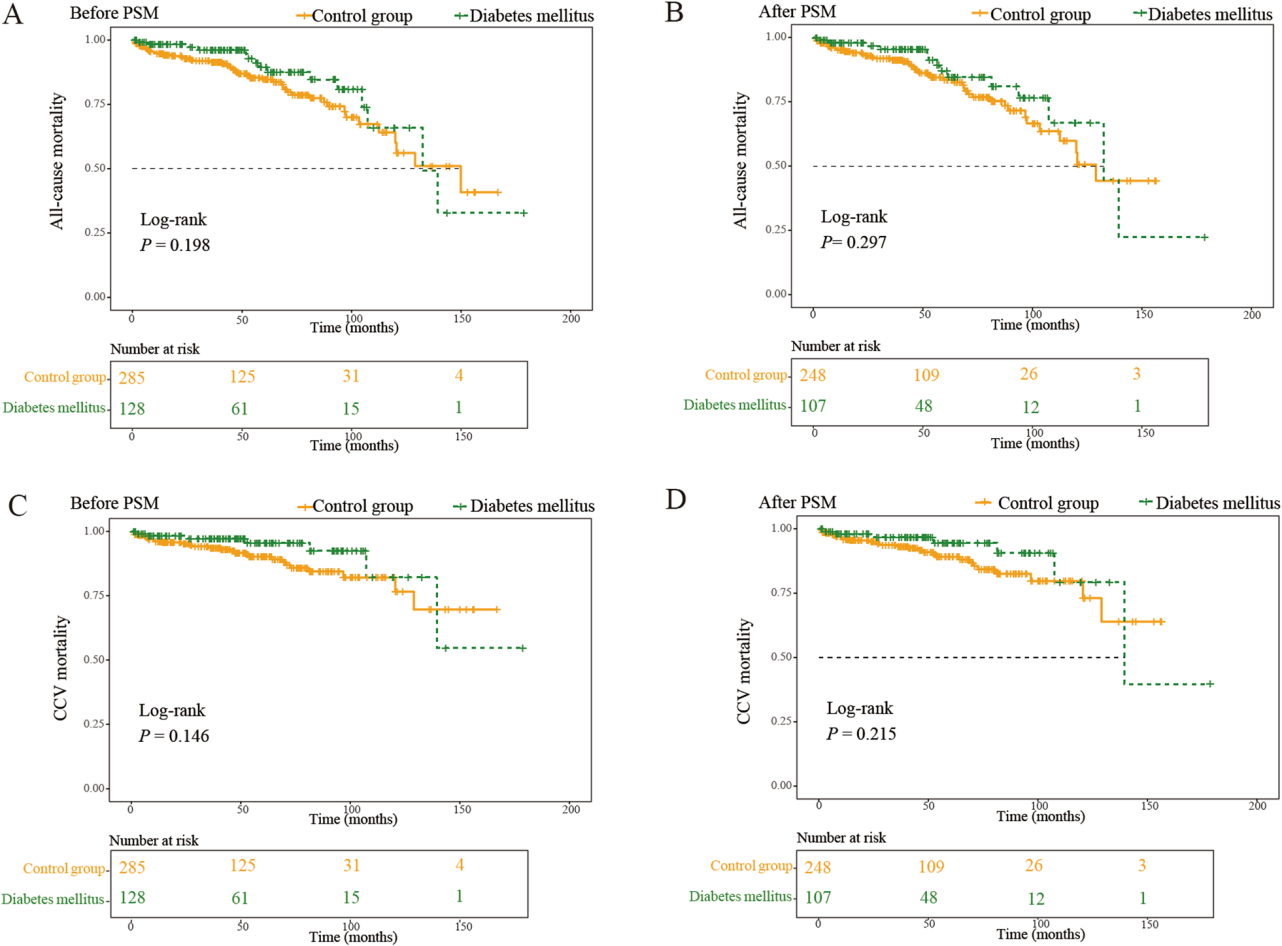
Impact of diabetes mellitus on long-term survival. Kaplan–Meier curves of all-cause mortality before (**A**) and after (**B**) propensity score matching. Kaplan–Meier curves of cardio-cerebrovascular mortality before (**C**) and after (**D**) propensity score matching

The 413 patients were followed up from 1 to 178.6 months after CABG (median 46.2 months). The 1-, 3-, 5- and 10-year survival rates in the total cohort were 95.8%, 93.2%, 86.1% and 61.6%, respectively. The long-term mortality in the elderly after CABG in this research was mainly caused by cardio-cerebrovascular events (55.6%), malignant tumor (15.9%), and other diseases (28.6%) such as infection, renal failure, and fracture. The 1-, 3-, 5- and 10-year survival rates in the overweight and obesity group versus the normal weight and lean group were 95.8% vs. 95.8%, 94.5% vs. 91.9%, 90.3% vs. 82.4%, and 74.5% vs.52.6%, respectively. The long-term postoperative survival was evidently improved in the overweight and obesity group compared to the normal weight and lean group. The Kaplan–Meier analysis by log-rank test showed that the overweight and obese patients had a lower incidence of all-cause death than the normal weight and lean group before and after PSM (Fig. 3A, B). Furthermore, overweight and obesity was associated with a significantly lower risk of long-term CCV mortality after CABG before and after PSM (Fig. 3C, D). Neither long-term all-cause mortality (Fig. 4A, B) nor CCV mortality (Fig. 4C, D) after CABG was significantly influenced by DM during the follow-up.

The COX proportional model suggested that before PSM, overweight and obesity (HR = 0.501, 95% CI 0.288–0.871, *P* = 0.014) and preoperative Scr (HR = 1.010, 95% CI 1.004–1.015, *P *< 0.001) impact long-term prognosis. After PSM, overweight and obesity still presented to be a protective factor to the long-term postoperative survival with significant importance (HR = 0.512, 95% CI 0.279–0.939, *P* = 0.030) (Table 3). Besides, with multivariate cox regression analysis, overweight and obesity was independent protective factor for long-term CCV mortality before (HR = 0.391, 95% CI 0.179–0.853, *P* = 0.018) and after PSM (HR = 0.431, 95% CI 0.191–0.972, *P* = 0.042) (Additional file 1: Table S1).

**Table 3:** Independent risk factors of long-term mortality by multivariate cox regression analysis before and after matching

	Before PSM		After PSM	
	Multivariate Cox regression		Multivariate Cox regression	
	HR (95% CI)	*P* value	HR (95% CI)	*P* value
Age	0.941 (0.830; 1.067)	0.346	0.982 (0.859; 1.122)	0.786
Male sex	0.437 (0.186; 1.028)	0.059	0.426 (0.167; 1.086)	0.074
Overweight and Obesity	0.501 (0.288; 0.871)	0.014	0.512 (0.279; 0.939)	0.030
Diabetes mellitus	0.685 (0.374; 1.252)	0.219	0.753 (0.402; 1.411)	0.375
Hypertension	1.461 (0.809; 2.640)	0.209	1.565 (0.842; 2.908)	0.156
Cerebrovascular disease	1.372 (0.928; 2.029)	0.113	1.354 (0.890; 2.060)	0.157
NYHA classification	1.569 (0.991; 2.486)	0.055	1.504 (0.920; 2.461)	0.104
Types of CAD	0.915 (0.580; 1.445)	0.704	0.867 (0.538; 1.396)	0.556
Preoperative Scr	1.010 (1.004; 1.015)	< 0.001	1.009 (1.004; 1.015)	< 0.001
Peripheral vascular disease	0.361 (0.079; 1.657)	0.190	0.349 (0.073; 1.662)	0.186
Pulmonary hypertension	0.768 (0.506; 1.166)	0.215	0.827 (0.545; 1.254)	0.370
Preoperative atrial fibrillation	0.898 (0.248; 3.251)	0.870	0.901 (0.244; 3.323)	0.875
COPD	1.708 (0.697; 4.183)	0.242	1.726 (0.684; 4.357)	0.248
Previous PCI	1.064 (0.417; 2.718)	0.897	1.141 (0.426; 3.058)	0.793
Valvular disease	1.329 (0.570; 3.099)	0.510	1.546 (0.655; 3.649)	0.320
Coronary artery bypass grafts	0.921 (0.687; 1.235)	0.582	0.956 (0.697; 1.312)	0.783

*PSM* propensity score matching, *HR* hazard ratio, *NYHA* New York heart association, *CAD* coronary artery disease, *Scr* serum creatinine, *COPD* chronic obstructive pulmonary disease, *PCI* percutaneous coronary intervention

## Discussion

This study presents the potential effects of some metabolic diseases in elderly patients on the short- and long-term mortality after CABG. The baseline characteristics were balanced with the PSM approach to reduce the confounding bias. Despite no difference in short-term mortality, the long-term postoperative survival rate of overweight and obese patients was satisfactory.

With the improvement of economy and living standards in China, the dietary frameworks and lifestyle in the elderly have changed greatly. As evident risk factors for CADs, metabolic disorders such as DM and obesity increase markedly in the elderly [9, 10]. Due to the characteristics of Chinese race and eating habits, BMI ≥ 24 and ≥ 28 kg/m^2^ are defined as overweight and obesity respectively [11]. High BMI is related to high rate of death in patients up to age 75 years [12]. Previous researches showed that obesity could significantly increase the occurrence and development of CADs, and indicated the poor prognosis with increased all-cause mortality [10, 13]. Moreover, higher BMI is associated with higher rates of thromboembolism, wound infections and blood loss perioperatively, so as to increase the in-hospital duration and the death rate after cardiac surgeries [14–16]. However, further in-depth studies showed the opposite results [17–19]. Researchers have found the beneficial impact of overweight and obesity on the prognosis of CABG [20, 21], which is called the “obesity paradox”. However, most of these studies focused on the prognosis of white populations after cardiac surgeries at all ages. In our study, the patients ≥ 75 years old after CABG in China were investigated. We found that overweight and obese patients aged ≥ 75 years have better long-term prognosis of CABG with significantly lower all-cause and CCV mortality.

Some researchers hold the “obesity paradox” results from confounding bias [22, 23]. Sarcopenia, lower basal metabolic rate, metabolic disorders, and intake of multiple drugs complicate the obesity in the elderly [5, 24], which could influence the long-term outcome after CABG among the patients with CADs. However, after reducing the confounding bias by PSM approach, the protective effect of obesity and overweight on long-term survival after CABG in the elderly was similarly evident. Another putative mechanism states that the decreased mortality in obese patients is due to higher muscle mass [25]. Noticeably, higher BMI cannot directly indicate the body composition and fat distribution. Excessive BMI does not equal to the exceeding adipose tissues. The relatively low BMI in elderly patients often related to less muscle tissue, indicating reduced motor function and frailty, which is not conducive to the recovery and long-term survival after the surgery [26]. Individuals with higher BMI have higher caloric reserves in response to metabolic stress, which may explain the “obesity paradox” [27]. Thus, when determining obesity in the elderly, researchers should consider more indicators such as waist circumference and waist-hip ratio, so as to make a reasonable and effective decision as far as possible.

Overweight and obesity was independent protective factor for long-term CCV mortality after CABG in the elderly patients in our study. Some researchers have found the lower sympathetic activation in obese patients, which may decrease the prolonged negative effect to the cardio-cerebrovascular system and reduce CCV mortality [28]. Adipokines with anti-inflammation and anti-apoptosis abilities are reportedly a protective factor of cardio-vasculature, and may partly account for the “obesity paradox” [29]. Moreover, the higher brain natriuretic peptide in the obese elderly may also partly serve as a protective factor in the cardio-cerebrovascular system [30]. The "obesity paradox" lead us to reconsider the secondary prevention and treatment of CADs, which indicates that excessive weight-loss may be harmful to long-term prognosis after CABG.

DM was reportedly an independent risk factor for the incidence of adverse CADs [31]. DM patients have worse outcome after cardiac surgeries, due to higher sympathetic and renin-angiotensin activation, myocardial apoptosis and fibrosis, coronary endothelium inflammation [32]. Studies have proved the adverse effect of DM on the survival after PCI or CABG [33, 34]. In this research, the relationship between DM and 30-day postoperative mortality was similarly explicit. However, the disadvantage of DM was not observed in the long-term survival after CABG. The first detection of DM in most patients was before CABG and the disease was never properly treated with much health risks. With the diagnosis of DM, patients were treated with good compliance, which may reduce the risk of the disease. Furthermore, the long-term survival after CABG was not significantly related to DM, which potentially indicates the remaining advantage in selecting CABG as a standard treatment for elderly CAD patients with DM.

There are some limitations in this study. Firstly, as a retrospective observational study, some bias cannot be eliminated whether PSM was applied or not. Secondly, patients were selected from two hospitals, and the inclusion period was long. Hence, changes in perioperative treatment and care may affect the statistical analysis. Additionally, we did not investigate the effect of adipose metabolism on the survival of CABG, and prognostic factors such as procedural characteristics (left internal mammary artery graft, complete revascularization, etc.) and medications (aspirin, statin, etc.) were not included in the study, which will be included in our further study.

## Conclusions

In this retrospective study, the increased BMI in patients aged ≥ 75 was related to lower long-term all-cause or CCV mortality after isolated CABG. DM was associated with higher 30-day mortality in elderly patients, but not significantly related with the long-term survival after CABG.

## Supplementary Information

**Additional file 1. Table S1**: Independent risk factors of CCV mortality by multivariate cox regression analysis before and after matching.

**Additional file 2. Fig. S1**: Standardized differences for each variable before and after PSM. The absolute standardized differences < 0.2 indicate balanced matching. Y axis represents the baseline of variables. X axis represents the standardized mean difference value. PSM, propensity score matching; EuroSCORE II, European system for cardiac operative risk evaluation II; IABP, intra-aortic balloon pump; PCI, percutaneous coronary intervention; COPD, chronic obstructive pulmonary disease; Scr, serum creatinine; LVEF, left ventricular ejection fraction; NYHA, New York heart association; CAD, coronary artery disease.

## Data Availability

The datasets generated and/or analyzed during the current study are not publicly available due legal/ethical reasons but are available from the corresponding author on reasonable request.

## Abbreviations

DM: Diabetes mellitus
CABG: Coronary artery bypass grafting
BMI: Body mass index
PSM: Propensity score matching
JSPH: Jiangsu Province Hospital
SHCH: Shanghai Chest Hospital
CADs: Coronary artery diseases
CVDs: Cerebrovascular diseases
CCV mortality: Cardio-cerebrovascular mortality
COPD: Chronic pulmonary disease
EuroSCORE II: European System for Cardiac Operative Risk Evaluation II
NYHA: New York Heart Association
K–S: Kolmogorov–Smirnov
SD: Standard deviation
ANOVA: Analysis of variance
Scr: Serum creatinine
LVEF: Left ventricular ejection fraction
ORs: Odds ratios
CIs: Confidence intervals
HR: Hazard ratios
IABP: Intra-aortic balloon pump
