# Supplementary material for: Impacts of metabolic disorders on short- and long-term mortality after coronary artery surgery in the elderly
Source: BMC Cardiovasc Disord. 2022 Nov 24;22:504. doi: 10.1186/s12872-022-02954-6 (PMC9700925; doi:10.1186/s12872-022-02954-6)
Supplement: Supplementary file 1 — Additional file 1. Table S1: Independent risk factors of CCV mortality by multivariate cox regression analysis before and after matching. [file 12872_2022_2954_MOESM1_ESM.docx]

Supp.Table1. Independent risk factors of CCV mortality by multivariate cox regression analysis

before and after matching

|  | Before PSM | |  | After PSM | |
| --- | --- | --- | --- | --- | --- |
|  | Multivariate Cox regression | |  | Multivariate Cox regression | |
|  | HR (95%CI) | *P* value |  | HR (95%CI) | *P* value |
| Age | 0.974(0.835-1.137) | 0.742 |  | 1.011(0.858-1.192) | 0.892 |
| Male sex | 0.458(0.146-1.438) | 0.181 |  | 0.472(0.146-1.523) | 0.209 |
| Overweight and Obese | 0.391(0.179-0.853) | 0.018 |  | 0.431(0.191-0.972) | 0.042 |
| Diabetes mellitus | 0.632(0.268-1.491) | 0.295 |  | 0.717(0.300-1.711) | 0.453 |
| Hypertension | 1.017(0.486-2.131) | 0.964 |  | 0.957(0.452-2.026) | 0.908 |
| Cerebrovascular disease | 1.189(0.672-2.102) | 0.552 |  | 1.141(0.628-2.074) | 0.665 |
| NYHA classification | 1.069(0.575-1.984) | 0.834 |  | 1.129(0.595-2.140) | 0.710 |
| Types of CAD | 1.059(0.594-1.890) | 0.846 |  | 0.994(0.554-1.785) | 0.985 |
| Preoperative Scr | 1.002(0.993-1.012) | 0.611 |  | 1.003(0.994-1.012) | 0.564 |
| Peripheral vascular disease | 0.226(0.027-1.917) | 0.173 |  | 0.238(0.027-4.958) | 0.193 |
| Pulmonary hypertension | 0.949(0.568-1.588) | 0.843 |  | 1.001(0.604-1.660) | 0.997 |
| Preoperative atrial fibrillation | 0.837(0.164-4.273) | 0.830 |  | 0.829(0.161-4.263) | 0.822 |
| COPD | 3.223(1.218-8.525) | 0.018 |  | 2.972(1.096 -8.060) | 0.032 |
| Previous PCI | 2.020(0.770-5.300) | 0.153 |  | 2.130(0.793-5.721) | 0.134 |
| Valvular disease | 1.438(0.462-4.473) | 0.530 |  | 1.599(0.516-4.958) | 0.416 |
| Coronary artery bypass grafts | 0.838(0.574-1.223) | 0.359 |  | 0.880(0.592-1.308) | 0.528 |

PSM, propensity score matching; HR, hazard ratio; NYHA, New York heart association; CAD, coronary artery disease; Scr, serum creatinine; COPD, chronic obstructive pulmonary disease; PCI, percutaneous coronary intervention.
