# Supplementary material for: Impacts of metabolic disorders on short- and long-term mortality after coronary artery surgery in the elderly
Source: BMC Cardiovasc Disord. 2022 Nov 24;22:504. doi: 10.1186/s12872-022-02954-6 (PMC9700925; doi:10.1186/s12872-022-02954-6)
Supplement: Supplementary file 2 — Additional file 2. Fig. S1: Standardized differences for each variable before and after PSM. The absolute standardized differences < 0.2 indicate balanced matching. Y axis represents the baseline of variables. X axis represents the standardized mean difference value. PSM, propensity score matching; EuroSCORE II, European system for cardiac operative risk evaluation II; IABP, intra-aortic balloon pump; PCI, percutaneous coronary intervention; COPD, chronic obstructive pulmonary disease; Scr, serum creatinine; LVEF, left ventricular ejection fraction; NYHA, New York heart association; CAD, coronary artery disease. [file 12872_2022_2954_MOESM2_ESM.pdf]

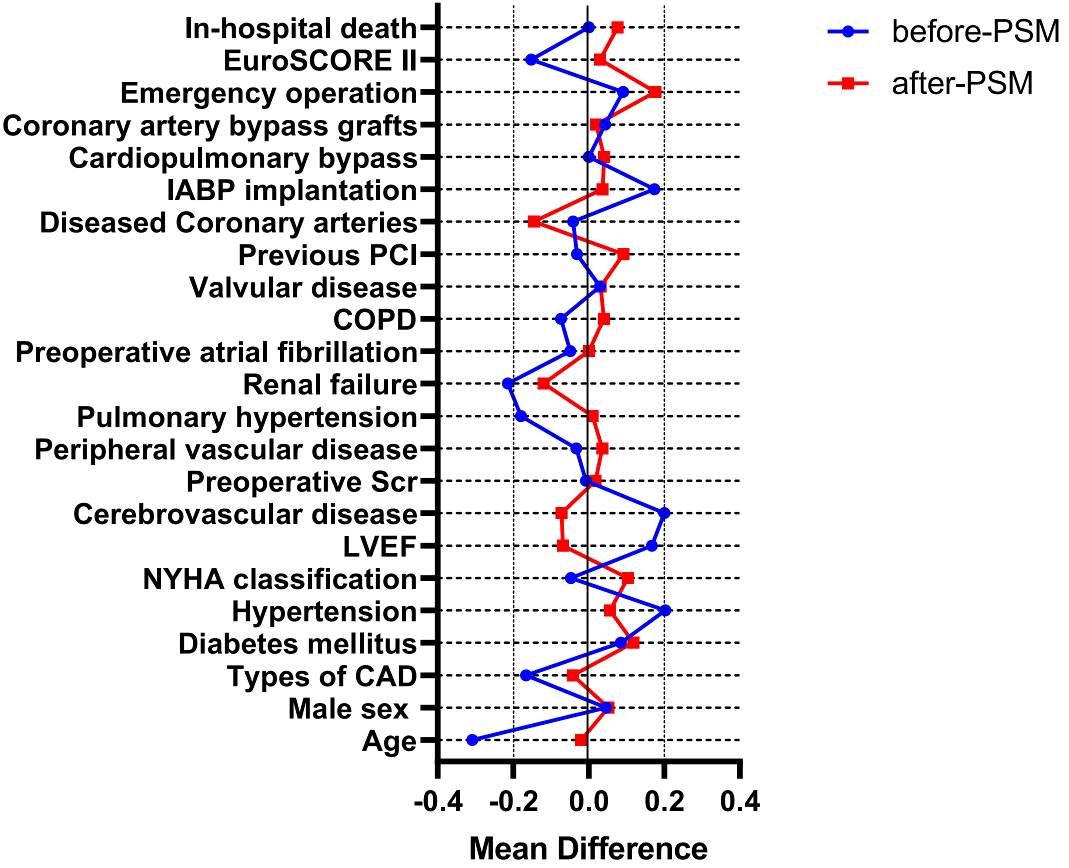

Suppl.Fig 1. Standardized differences for each variable before and after PSM. The absolute standardized differences <0.2 indicate balanced matching. Y axis represents the baseline of variables. X axis represents the standardized mean difference value. PSM, propensity score matching; EuroSCORE II, European system for cardiac operative risk evaluation II; IABP, intra-aortic balloon pump; PCI, percutaneous coronary intervention; COPD, chronic obstructive pulmonary disease; Scr, serum creatinine; LVEF, left ventricular ejection fraction; NYHA, New York heart association; CAD, coronary artery disease.
